# The association between objectively-measured activity, sleep, call responsibilities, and burnout in a resident cohort

**DOI:** 10.1186/s12909-019-1592-0

**Published:** 2019-05-21

**Authors:** Ashley P. Marek, Rachel M. Nygaard, Ellen T. Liang, Nicholas S. Roetker, Mary DeLaquil, Sandy Gregorich, Chad J. Richardson, Joan M. Van Camp

**Affiliations:** 1 Department of Surgery, Hennepin Healthcare, 701 Park Ave Minneapolis, Hennepin, MN 55415 USA; 2 Chronic Disease Research Group, Minneapolis Medical Research Foundation, Minneapolis, MN USA; 3 Minneapolis Medical Research Foundation, Minneapolis, MN USA

**Keywords:** Exercise, Burnout, Resident training, Physician wellness, Fitness tracking

## Abstract

**Background:**

When compared to the general US working population, physicians are more likely to experience burnout and dissatisfaction with work-life balance. Our aim was to examine the association of objectively-measured sleep, activity, call load, and gender with reported resident burnout and wellness factors.

**Methods:**

Residents were recruited to wear activity tracker bands and complete interval blinded surveys.

**Results:**

Of the 30 residents recruited, 28 (93%) completed the study. Based on survey results, residents who reported high amounts of call reported equivalent levels of wellness factors to those who reported low call loads. There was no association between amount of call on training satisfaction, emotional exhaustion, self-reported burnout, or sleep quality. Analysis of sleep tracker data showed that there was no significant association with time in bed, time asleep, times awakened or sleep latency and call load or self-reported burnout. Female gender, however, was found to be associated with self-reported burnout. No significant associations were found between objectively-measured activity and burnout.

**Conclusions:**

Based on the results of our study, there was no association with burnout and objectively-measured sleep, call volume, or activity. Increased call demands had no negative association with training satisfaction or professional fulfillment. This would suggest that more hours worked does not necessarily equate to increased burnout.

## Background

Efforts to identify and manage burnout in the medical field have become an increasingly necessary mission. Physicians are more likely to experience burnout and dissatisfaction with work-life balance compared with the general U.S. working population [1]. Burnout is seen at the resident physician and even medical student level [2–4]. A survey of 753 general surgery residents found that 69% met criteria for burnout, with almost half of residents who reported burnout considering dropping out of their program [3, 5]. Dyrbye et al. showed that burnout was higher at the resident level when compared with physicians early in their career [6].

Existing studies indicate that insufficient sleep is a risk factor for subsequent burnout [7]. Not only does sufficient sleep improve the ability to cope with emotional challenges, lack of sleep may increase susceptibility to stress [8]. Residency training often includes in-house call, home call, and night float service obligations that may disrupt sleep [9]. Studies examining the impact of sleep on burnout or quality of life largely rely on self-reported measures.

In a cohort of surgical trainees, mental fitness training improved well-being [10]. Existing studies that examine resident and physician well-being, burnout, and activity are largely survey-based and descriptive only. Our aim was to examine the association of objectively-measured sleep, activity, call load, and gender with reported resident wellness factors and burnout.

## Methods

After obtaining IRB approval (HSR 15–4081) and informed consent, 30 emergency medicine and general surgery residents at a single institution were enrolled in the study. Funding allowed for 30 participants through purchase of tracker bands. Of the 30 residents recruited, 28 (93%) completed the study. One resident dropped out of the study for medical reasons and another resident did not tolerate wearing the fitness tracker and elected to withdraw from the study. Our IRB precluded us from collecting potentially-identifying information, specifically the resident’s training program, program year, marital status, or presence of children in the home. The tracking bands were linked to blinded accounts to de-identify survey and tracker data.

The participants wore activity tracker bands (Fitbit Charge HR™) over a 16-week study period. During the study, the participants completed blinded surveys with the number of in-house call and home call shifts worked, gender, height, weight, wellness factors (sleep quality, perceived appreciation, professional fulfillment and emotional exhaustion), and a validated single item burnout score. The wellness factors survey was obtained from the Linzer research group at our institution [11]. A validated single-item burnout measure was used for self-evaluation [12, 13]. We defined residents as not exhibiting burnout if they answered: (1) I have no symptoms of burnout or (2) Occasionally I am under stress, but I don’t feel burned out. We defined residents as having burnout symptoms if they answered: (3) I am definitely burning out and have one or more symptoms of burnout, (4) The symptoms of burnout that I’m experiencing won’t go away, or (5) I feel completely burned out and often wonder if I can go on. A resident reporting burnout was set to a score of ≥3. Call load was dichotomized as high or low based on institutional norms. High call load was categorized as greater than or equal to 7 days of in-house call or 24 days of home call in past 4 weeks.

The tracker bands measured resident activity. Exercise heart rate zone was defined as a combination of time in ‘fat burning’, ‘cardio’, and ‘peak’ heart rate zones, as measured and reported by the Fitbit Charge HR™ app. Active time was defined as a combination of ‘fairly active minutes’ and ‘very active minutes’, as measured and reported by the Fitbit Charge HR™ app. Other measurements included daily step count, time asleep, time awake in bed, times awakened from sleep, time in bed, and resting heart rate – all measured and reported by the Fitbit Charge HR™ app.

Statistical analyses were conducted using SAS 9.4 TS level 1 MS [SAS Institute Inc. Cary, NC], STATA 12.2 [StataCorp College Station, TX], and G*Power 3.1.9.4 [Heinrich Heine Universitat Dusseldorf]. Student T-test and Fisher’s exact were calculated for continuous and categorical variables, respectively. For the assessment of an association between self-reported burnout and activity levels, follow-up was divided into four study periods: weeks 1–3, weeks 4–8, weeks 9–13, and weeks 14–16. The exposure of interest, self-reported burnout (yes or no), was measured via a wellness survey at the beginning of each study period. The outcomes of interest were the average daily levels of each activity measured across each study period. Thus, burnout and activity levels were measured in a repeated fashion, up to four times. For each activity, the mean activity level was modeled as a function of burnout category, study period, treatment group, sex, and body mass index using a linear generalized estimating equation (GEE) model. An exchangeable working correlation structure was specified to account for correlation between repeated activity measurements over time. Given our sample size of 28, we could expect an effect size f = 0.548. The GEE model was used to estimate the mean difference in activity level between those with and without burnout over follow-up.

## Results

Of the 30 residents recruited, 28 (93%) completed the study. Half the residents were female and the average age was 30. In the initial survey prior to fitness tracker use, the average amount of in-house or home call was 6.4 days in the preceding 4 weeks. More females were in the high call group, but this difference was not statistically significant (Table 1). There was no association between the amount of call on training satisfaction, emotional exhaustion, or sleep quality. Differences in wellness factors between male and female residents were only observed in emotional exhaustion (2.5 vs 3.1, *P* = 0.043) and a non-significant trend toward higher interpersonal disengagement (2.2 vs 2.8, *P* = 0.075) (Table 1). At baseline, one-third of residents reported experiencing symptoms of burnout. While more female residents reported feeling symptoms of burnout (50% vs 14.3%, *P* = 0.103), the difference was not statistically significant (Table 1).

**Table 1:** Baseline survey and resident cohort^a^

	Cohort *N* = 28	Male *N* = 14	Female *N* = 14	*P* value^b^
Age, mean (SD)	30 (2)	30 (3)	30 (2)	0.876
In house call, N (%)				0.487
0	1 (3)	0 (0)	1 (7)	
1–5	7 (25)	5 (36)	2 (14)	
6–8	12 (43)	6 (43)	6 (43)	
9+	8 (29)	3 (21)	5 (36)	
Home call, N (%)				0.730
0	24 (86)	13 (93)	11 (79)	
1–5	1 (4)	0 (0)	1 (7)	
6–8	2 (7)	1 (7)	1 (7)	
9–19	0 (0)	0 (0)	0 (0)	
20+	1 (4)	0 (0)	1 (7)	
Call group high^c^, N (%)	16 (57.1)	6 (37.5)	10 (62.5)	0.252
Wellness factors^d^, mean (SD)				
Professional Fulfillment	2.7 (0.7)	2.5 (0.6)	2.9 (0.7)	0.119
Appreciation	3.0 (0.6)	2.8 (0.6)	3.1 (0.5)	0.308
Values Alignment	2.5 (0.6)	2.4 (0.7)	2.5 (0.5)	0.620
Training Satisfaction	2.2 (0.6)	2.3 (0.8)	2.1 (0.5)	0.328
Peer Support	2.3 (0.6)	2.4 (0.5)	2.2 (0.7)	0.482
Emotional Exhaustion	2.8 (0.7)	2.5 (0.5)	3.1 (0.8)	0.043
Interpersonal Disengagement	2.5 (0.8)	2.2 (0.8)	2.8 (0.8)	0.075
Negative Impact of Work on Personal Relationships	3.1 (1.0)	2.9 (1.0)	3.3 (1.1)	0.351
Sleep Quality	3.0 (0.8)	2.9 (0.7)	3.2 (1.0)	0.345
Self-reported Burnout Score, N (%) symptoms				0.089
2	19 (68)	12 (86)	7 (50)	
3	6 (21)	2 (14)	4 (29)	
4	3 (11)	0 (0)	3 (21)	
Self-reported Burnout^e^, N (%)	9 (32.1)	2 (14.3)	7 (50)	0.103

^a^Data from Survey 1

^b^Student T-test for continuous variables and Fisher’s exact for categorical variables

^c^High call load categorized as greater than or equal to 7 days of in house call or 24 days of home call in past 4 weeks

^d^Likert scale: values for each item were set to score ‘high burnout’ or a negative response as the highest value (5) and ‘low burnout’ or a positive response (1)

^e^Burnout describes self-reported burnout symptoms or a score of ≥3

We examined the association between call and self-reported burnout with sleep measured by the activity tracker (Table 2). Residents who reported high amounts of call reported a trend toward lower levels of appreciation (3.1 vs 2.6, *P* = 0.106), and a higher level of negative impact of work on personal relationships (3.4 vs 2.7, *P* = 0.161) than residents who had less call, however these differences failed to reach statistical significance (Table 2). There was no significant association between the amount of call and training satisfaction, emotional exhaustion, sleep quality, or self-reported burnout (Table 2). There was no significant association between the amount of call and measured amounts of sleep (Table 2). Female residents were more likely to report symptoms of burnout compared to male residents. There was no significant association between burnout and measured sleep. Interestingly, resting heart rate was significantly higher in residents experiencing burnout (Table 2).

**Table 2:** Impact of call on resident wellness factors, burnout, and sleep^a^

		Call			Self-reported Burnout		
	Cohort *N* = 28	High Call *N* = 11	Low Call *N* = 17	*P* value^b^	Symptoms *N* = 6	No symptoms *N* = 22	*P* value^b^
Gender F, N (%)	14 (50)	5 (46)	9 (53)	1.000	6 (100)	8 (36)	0.016
Call group high^c^, N (%)	11 (39.3)	–	–	–	3 (50)	8 (36)	0.653
Wellness factors^d^, mean (SD)							
Professional Fulfillment	2.6 (0.8)	2.6 (0.7)	2.6 (0.8)	0.857	–	–	–
Appreciation	2.8 (0.8)	3.1 (0.7)	2.6 (0.8)	0.106	–	–	–
Values Alignment	2.4 (0.9)	2.4 (0.5)	2.4 (0.9)	0.933	–	–	–
Training Satisfaction	2.1 (0.9)	2.2 (1.0)	2.0 (0.9)	0.607	–	–	–
Peer Support	2.1 (0.8)	2.2 (0.7)	2.0 (0.8)	0.776	–	–	–
Emotional Exhaustion	2.6 (0.7)	2.7 (0.7)	2.5 (0.7)	0.399	–	–	–
Interpersonal Disengagement	2.2 (0.7)	2.3 (0.7)	2.1 (0.7)	0.418	–	–	–
Negative Impact of Work on Personal Relationships	3.0 (1.1)	3.4 (0.9)	2.7 (1.2)	0.161	–	–	–
Sleep Quality	2.8 (0.8)	2.9 (0.9)	2.8 (0.8)	0.679	–	–	–
Self-reported Burnout group^e^, N (%)	6 (21.4)	3 (27)	3 (18)	0.653	–	–	–
Asleep (min), mean (SD)	362.4 (97.9)	386.6 (60.8)	346.8 (114.8)	0.303	391.3 (42.6)	354.5 (107.6)	0.424
Time awake (min) in bed, mean (SD)	23.3 (21.0)	19.2 (10.1)	26.0 (25.8)	0.415	20.3 (6.9)	24.1 (23.5)	0.700
Times awakened, N (%)	11.0 (4.9)	10.6 (5.4)	11.3 (4.7)	0.700	11.9 (4.5)	10.8 (5.1)	0.657
Time in bed (min), mean (SD)	387.1 (94.5)	406.6 (58.0)	374.5 (111.9)	0.391	412.7 (45.9)	380.1 (103.6)	0.464
Resting heart rate (bpm), mean (SD)	67.1 (6.2)	66.9 (5.5)	67.2 (6.7)	0.904	71.6 (3.6)	65.9 (6.2)	0.044

^a^Data tracked using Fitbit Charge HR™ and Survey 2

^b^Student T-test for continuous variables and Fischer’s Exact test for categorical variables

^c^High call load categorized as greater than or equal to 7 days of in house call or 24 days of home call in past 4 weeks

^d^Likert scale: values for each item were set to score ‘high burnout’ or a negative response as the highest value (5) and ‘low burnout’ or a positive response (1)

^e^Burnout describes self-reported burnout symptoms or a score of ≥3

Abbreviations: bpm, beats per minute; F, female; min, minutes; SD, standard deviation

**Fig. 1:**
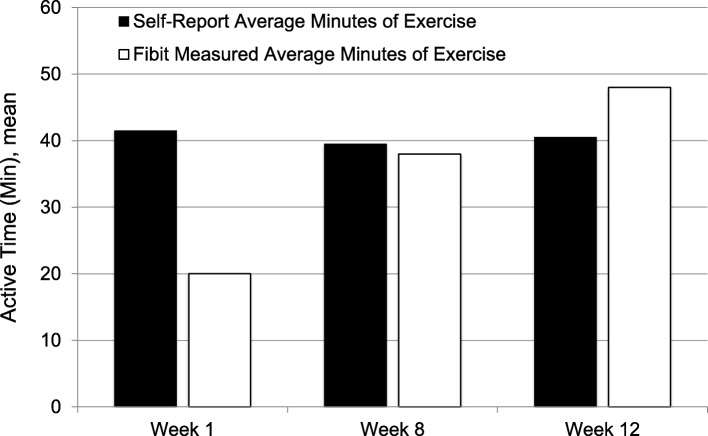
Self-reported and activity tracker measured activity levels of participating residents on weeks 1, 8, 12

We also examined the association between overall activity and self-reported burnout (Table 3). We found no significant differences in activity level between those with and without burnout in this cohort of residents (Table 3). Throughout the study the residents were also asked to self report the amount of exercise they engaged over the previous 4 weeks. We then compared this with the measured activity level from the activity tracker band. The initial self-reported activity was half of the measured activity (Fig. 1). This discrepancy minimized over the course of the study with further use of the activity tracker band.

**Table 3:** Association between self-reported burnout and activity levels

	Daily mean ± SE across all study periods		Difference (95% CI) between burnout groups	*P*-value^a^
Activity measure	No burnout	Burnout		
Exercise HR Zone^b^, min	142 ± 16	120 ± 13	−22 (−50 to 6)	0.12
Active Time^c^, min	40 ± 6	40 ± 7	−0.1 (−13 to 13)	0.99
Steps	9690 ± 380	9833 ± 482	143 (− 694 to 980)	0.74

^a^The mean activity level was modeled as a function of burnout category, study period, treatment group, sex, and body mass index using a linear generalized estimating equation (GEE) model. An exchangeable working correlation structure was specified to account for correlation between repeated activity measurements over time. The GEE model was used to estimate the mean difference in activity level between those with and without burnout over follow-up

^b^Exercise heart rate zone was defined as a combination of time in ‘fat burning’, ‘cardio’, and ‘peak’ heart rate zones, as measured and reported by the Fitbit Charge HR™ app

^c^Active time was defined as a combination of ‘fairly active minutes’ and ‘very active minutes’, as measured and reported by the Fitbit Charge HR™ app

Abbreviations: CI, 95% confidence interval; HR, heart rate; min, minutes; SE, standard error

## Discussion

The stressors of residency training, which are multifactorial, may lead to high levels of burnout among residents. In this study, 32% of residents reported some symptom of burnout. Female residents reported feeling symptoms of burnout more frequently than their male counterparts. Neither measured sleep nor call volume was associated with higher rates of burnout. There was no association between high call load and professional fulfillment or training satisfaction. Activity level was not associated with burnout in this cohort of residents.

Studies examining the influence of gender on burnout and quality of life factors have showed mixed results in the U.S. physician population [14–17]. In a comparison of medical students, residents, and early career physicians to the general U.S. population, burnout was not significantly different between males and females; however, male physicians and trainees were significantly less likely to report high fatigue or exhibit signs of depression [6]. In our cohort of residents, females consistently reported higher rates of burnout than their male counterparts.

In a resident survey, 40% reported their sleep needs were not met on call; however, there were no differences in well-being noted when compared to residents whose sleep needs while on call were generally met [18]. In our study, both time in bed and time asleep was slightly higher in the high call group, though this difference did not reach statistical significance. Residents in our study who reported higher amounts of call did not report more burnout than residents with fewer call responsibilities. Of interest, there was also no association between the amount of call with professional fulfillment or training satisfaction. This is in alignment with studies of residents’ perceptions after the 2011 duty hour restrictions [19]. In a survey of surgery residents, it was felt the new rules worsened continuity of care and decreased time in the operating room, which led to concerns about preparedness after graduation [20].

Based on the subjective findings of sleep and burnout, one could infer that decreased time spent working should improve sleep and therefore wellness and burnout. Lindeman et al. surveyed general surgery residents before and after the 2011 duty hour rules were implemented and found that while burnout had indeed decreased from 93 to 75%, there was no significant change reported in sleepiness [21]. Other studies have shown no difference in resident burnout or quality of life after the duty hour change [18, 20]. In our study, the residents’ year of training was blinded to the researchers, so we cannot comment on whether interns demonstrated differences in burnout or wellness when compared to the more senior residents. However, when sleep was measured using the tracking band, there was no association between burnout and sleep. Perhaps, despite no difference in actual sleep time as measured by the activity band, a perceived lack of sleep contributes to burnout symptoms or vice versa.

The mere act of wearing an activity tracker may have impacted our residents’ perception of well-being. In a study of emergency medicine residents questioned prior to and after wearing activity tracker bands, there were subjective improvements in overall wellness without increases in measured physical activity [22]. Interestingly, we found no significant association between measured activity and burnout. Studies that describe resident exercise habits are largely descriptive and based on self-reported surveys. A survey-based study showed that 73% of residents meet the US Department of Health and Human Services recommendation of 150 min of moderate-intensity aerobic activity and 75 min of vigorous-intensity aerobic activity per week [23]. Our residents over-reported their exercise times compared to their activity tracker measurements.

This study has the typical limitations associated with single-institution studies and a small sample size. There are many variables we were unable to control for during the study that may have contributed to activity levels. While there were emergency medicine and general surgery residents participating in the study, we were unable to determine the make-up of the two groups by specialty given the limitations of our IRB. It is possible that any differences in the programs may have influenced the differences observed between groups. The ability of the tracker band to measure activity, while fairly accurate, is imperfect, though our ability to measure these outcomes will improve as affordable personal trackers continue to evolve. Future studies could improve on our pilot study by increasing the sample size with residents from multiple training programs at multiple institutions.

## Conclusions

Based on the results of our study, there was no association with burnout and objectively-measured sleep, call volume, or activity. Increased call demands had no negative association with training satisfaction or professional fulfillment. This would suggest that more hours worked does not necessarily equate to increased burnout. It is important to identify factors in resident training to mitigate burnout and promote a healthy life-long career in medicine.
